# Optimization of PBFT Algorithm Based on QoS-Aware Trust Service Evaluation

**DOI:** 10.3390/s22124590

**Published:** 2022-06-17

**Authors:** Wei Liu, Xuhao Zhang, Wenlong Feng, Mengxing Huang, Yun Xu

**Affiliations:** 1 School of Information and Communication Engineering, Hainan University, Haikou 570228, China; liuwei@hainmc.edu.cn (W.L.); zxhchinazgyd@163.com (X.Z.); 2 College of Biomedical Information and Engineering, Hainan Medical University, Haikou 571199, China; 3 Department of Software Engineering, Wenchang Satellite Launch Center, Wenchang 571300, China; xu.yun001@stu.xjtu.edu.cn

**Keywords:** QoS-aware, blockchain, consensus mechanism, PBFT

## Abstract

In service-transaction scenarios, blockchain technology is widely used as an effective tool for establishing trust between service providers and consumers. The consensus algorithm is the core technology of blockchain. However, existing consensus algorithms, such as the practical Byzantine fault tolerance (PBFT) algorithm, still suffer from high resource consumption and latency. To solve this problem, in this study, we propose an improved PBFT blockchain consensus algorithm based on quality of service (QoS)-aware trust service evaluation for secure and efficient service transactions. The proposed algorithm, called the QoS-aware trust practical Byzantine fault tolerance (QTPBFT) algorithm, efficiently achieves consensus, significantly reduces resource consumption, and enhances consensus efficiency. QTPBFT incorporates a QoS-aware trust service global evaluation mechanism that implements service reliability ranking by conducting a dynamic evaluation according to the real-time performance of the services. To reduce the traffic of the blockchain, it uses a mechanism that selects nodes with higher values to form a consensus group that votes for consensus according to the global evaluation result of the trust service. A practical protocol is also constructed for the proposed algorithm. The results of extensive simulations and comparison with other schemes verify the efficacy and efficiency of the proposed scheme.

## 1. Introduction

In service-transaction scenarios, service providers encapsulate resources (such as software and data) into services for publishing and sales. Service consumers can search for, purchase, and use services to meet their needs. However, traditional service-trading platforms typically lack trust mechanisms and have insufficient security protection. In recent years, several methods have been proposed for introducing blockchain technology, which was initially launched with the Bitcoin whitepaper by Satoshi Nakamoto in 2008 [1], into service transactions in an attempt to solve the security problems and trust deficits in the process of service transactions. Blockchain technology provides distributed shared ledgers and databases that have salient features, such as decentralization, tampering-proofing, full traceability retention, suitability, collective maintenance, robustness, and transparency. These ledgers and databases are shared through decentralized peer-to-peer exchange within a network. All nodes in the blockchain form a peer-to-peer network and all participating nodes are equal and collaboratively provide services. Without a central service node, the risk of a single bottleneck point is reduced. Nowadays, blockchain is widely used in fields such as finance [2], supply chain [3], energy trading [4], healthcare [5], Internet of Things [6], and other fields [7,8].

The consensus algorithm is the most important part of the blockchain as it establishes flexible trust relationships in distributed systems and affects the security and efficiency of blockchain products. The most commonly used consensus algorithms are the proof-of-work (PoW), proof-of-stake (PoS), delegated proof-of-stake (DPoS), practical Byzantine fault tolerance (PBFT), Raft, and proof-of-authority (PoA) algorithms. Among these algorithms, PoW, PoS, and DPoS are widely used in public blockchain applications. The PBFT algorithm is primarily used for consortium blockchains to solve general Byzantine problems. The Raft algorithm is generally considered a consensus algorithm for private blockchains and is suitable for more ad hoc networks, such as intranets. As a consensus mechanism in consortium blockchain, PBFT has the advantages of high efficiency and fast feedback. However, PBFT still suffers from high resource consumption and latency, which is not efficient when the distributed system is scaled up. The communication costs necessary to form a consensus in the mesh network increase exponentially as the number of nodes increases in the PBFT system, resulting in a scalability problem. To solve this problem, in this study, we propose an improved PBFT blockchain consensus mechanism based on QoS-aware trust service evaluation for secure and efficient service transactions called the QoS-aware trust practical Byzantine fault tolerance (QTPBFT) algorithm.

In summary, the main contributions of this study are as follows.

(1) We propose a QoS-aware trust service global evaluation mechanism to achieve reliability ranking of services. Services with higher evaluation values are considered more reliable and secure. The global evaluation is calculated by integrating the performance of static and dynamic QoS, where the static QoS value is the initial state of the service, which is provided by the service provider, and the dynamic QoS value is the real-time performance of the service, which is captured by monitoring the QoS parameters of the service.
(2) We develope an improved PBFT consensus algorithm called QTPBFT that is based on the trust service global evaluation mechanism. The QTPBFT algorithm introduces a mechanism to select nodes participating in the consensus based on their QoS-aware trust value, which reduces the communication cost in the network. Nodes with higher degree of trust are selected to form the main consensus group.
(3) We constructe a practical protocol for the proposed system. Simulation experiments and analysis of the optimization scheme verify its efficacy and efficiency.

## 2. Related Work

In this section, we discuss related work in terms of the basic PBFT consensus scheme and improved PBFT based on blockchain.

### 2.1. The Basic PBFT

PBFT is a consensus algorithm introduced in 1999 by Liskov and Castro [9]. It provides a practical Byzantine state that works even when malicious nodes are running in the system, and is one of the most popular mechanisms for protecting distributed systems from malicious users. The core theory underlying PBFT is formulated as Equation (1).

(1) n≥3f+1

where *n* is the total number of nodes in the system and *f* is the number of malicious nodes. In other words, if the system allows *f* malicious nodes, then the system must have *n* nodes to reach a consensus regarding the state of the system using the majority rule. 3f+1 is the minimum number of replicas that allows an asynchronous system to provide safety and liveness properties. The system becomes more secure as the number of nodes *n* increases.

The operation of the PBFT system requires at least four participants, one of which is elected as the primary node (or the leader node), and the other three are referred to as secondary nodes (or backup nodes). All nodes in the system communicate with each other, aiming to reach a consensus based on the principle of the minority obeying the majority. If the primary node is lying, the other nodes can join forces to replace it. The PBFT consensus algorithm typically consists of the following four steps:

- Step 1: Request. The client sends a request to the primary (leader) to perform an operation.
- Step 2: Pre-prepare. The primary (leader) node broadcasts the request to each secondary (backup) node.
- Step 3: Prepare. After receiving the preparation message, and after confirming that the information is correct, all nodes (primary and secondary) verify the message, execute the request, and then send a reply to the client.
- Step 4: Commit. When the client receives f+1 identical replies from different nodes, the process ends, where *f* is the maximum number of faulty nodes allowed.

**Figure 1:**
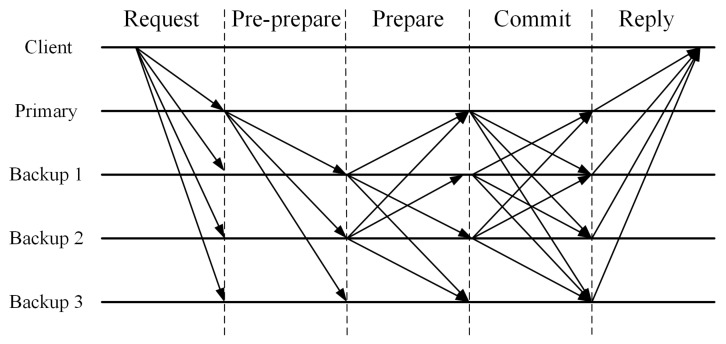
The operation of the PBFT algorithm.

Figure 1 shows the operation of the algorithm in the normal case of no primary faults. Owing to its low complexity and resource consumption, it is favored by consortium blockchains. However, the PBFT consensus works efficiently only when the number of nodes in the distributed network is small; the efficiency falls sharply with the expansion of the network scale because the communication overhead increases exponentially with every extra node in the network, and there is also the problem of low scalability.

### 2.2. The Improved PBFT

Many PBFT variants have been proposed that purportedly improve the quality and performance of PBFT for specific cases and conditions. Some researchers have proposed adding cryptographic mechanisms and algorithmic optimizations to PBFT. One of the most famous methods is Zyzzyva [10], which adds many encryption mechanisms to improve the security of the protocol by verifying the identity of clients to replicas and replicas to each other. Zyzzyva speculates that all replicas are correct, so they can process requests and send replies to the client without communicating with each other. There is no message exchange between replicas. The protocol performs differently in different situations. In the fast situation, that is, all replicas are honest, consensus can be reached in a very short time. However, in other situations, if there are any bad replicas, a complex recovery phase is initiated and recovery time is required; therefore, performance may deteriorate significantly, and the consensus will be slower than PBFT. SBFT [11] combines protocol steps with threshold cryptography and extends Zyzzyva by introducing communication and execution nodes called collectors. In addition, other BFT blockchain consensus algorithms have been proposed to improve PBFT, such as dBFT [12] and Ouroboros-BFT [13]. However, these consensus algorithms still have problems of low efficiency or low scalability, and some algorithms have a strong dependence on the primary node in consortium blockchains.

Researchers have also proposed additional mechanisms to improve the robustness of the PBFT algorithm. In redundant BFT (RBFT) [14], reliability is achieved through resource redundancy because each node runs f+1 PBFT protocol instances, including 1 primary and *f* replicas. All the instances sort the requests, but only the requests sorted by the primary instance are executed. Other instances are backups for throughput measurement. If the measured throughput of the primary instance is lower than that of the best backup instance, the view should be changed. RBFT adds the concept of random numbers, which weakens the authority of the master node. However, it has little improvement over the PBFT algorithm and its scope of application is limited. Whenever the primary node broadcasts information, it takes too much time for consensus nodes to verify the response. In FairLedger [15], which is a permissioned blockchain BFT protocol, a communication abstraction protocol, called Detectable All-to-All (DA2A), detects participants who deviate from the protocol (Byzantine or rational) and punishes them. T-PBFT [16], which is a multi-stage consensus algorithm, is an optimized PBFT consensus algorithm based on the EigenTrust model that replaces a single primary node with a primary group. X-layer PBFT [17] incorporates a scalable multilayer PBFT-based consensus mechanism that hierarchically groups nodes into different layers and limits the communication within the group. It extends the optimal double-layer PBFT to arbitrary-layer PBFT systems. However, most of these methods are focused on fault tolerance and scalability, and consensus efficiency and dynamically changing node states are rarely considered.

From the above analysis, it is clear that much work has been done to improve the PBFT algorithm. The methods proposed reduce the scale of the consensus cluster from different perspectives and improve the consensus efficiency of the PBFT algorithm to a certain extent. However, there are limitations in applying these consensus algorithms to blockchain-based service-trading platforms. Although the algorithms proposed above involve improvements in terms of resource consumption, throughput, and latency, there is still room for optimization. In our previous study [18], a PBFT-optimized algorithm based on an improved C4.5 was proposed. In a service-transaction scenario, based on the initial static QoS and real-time dynamic QoS parameters of the service, this study proposes a global evaluation scheme for further optimization.

## 3. Qos-Aware Trust Service Evaluation

Service trust value is an essential element in establishing trust among multiple parties in online electronic transactions, particularly in open public networks. Nodes participating in a transaction should provide trusted identity information and establish secure communications between multiple parties. Algorithmic or mathematical modeling methods can be used to measure the trustworthiness of a service provider’s promises to its customers. Particularly in a distributed service environment, a suitable service trust evaluation solution is an effective way to solve service security problems, and it is essential for service providers and customers to protect their rights.

### 3.1. Overview of Evaluation Strategy

**Figure 2:**
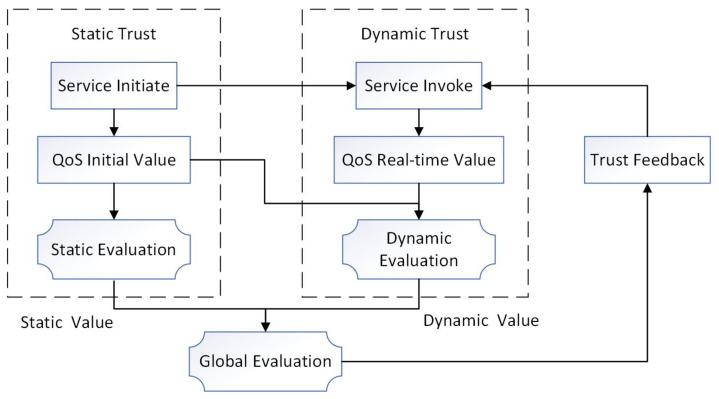
The overall framework of the service trust evaluation strategy.

In this paper, we propose a QoS-aware trust service global evaluation scheme, which is calculated from the static initial value of the service and dynamic value that reflects real-time service status. The overall framework of the service trust global evaluation strategy is illustrated in Figure 2.

The service trust evaluation scheme comprises two parts: static and dynamic trust. Initially, static evaluation is calculated based on the service’s initial QoS value provided by the provider. The dynamic evaluation is then calculated according to the real-time QoS value obtained from the monitoring parameters after the service invocation. To reduce the abnormality and distortion of real-time parameters, it is appropriate to monitor for some time and average the parameters. Finally, global evaluation is calculated by integrating the static and dynamic values, which are fed back to the consumer as the trust parameter of the service. The global evaluation values are sorted from high to low. Those parameters with higher scores are considered more credible and can form a consensus group to participate in the blockchain consensus process.

### 3.2. Detailed Evaluation Process

The proposed scheme utilizes static analysis and dynamic analysis in combination to form a specific global evaluation system, which results in the evaluation of service quality. To evaluate QoS based on non-functional indicators of the service, this study utilizes the response time (RT), throughput (TP), successability (SA), best practices (BP), and latency (LC) as evaluation indicators. The format is as Equation (2).

(2) $$\begin{array}{c} QoS=\{RT,TP,SA,BP,LC\} \end{array}$$

The detailed global evaluation process comprises the following five steps.

- Step 1: Set QoS parameters of provider’s service.
  In this study, QoS parameters are set in the form of intervals. The format is as Equation (3).
  (3) $$a=\left[\begin{array}{cc} a^{-}, & a^{+} \end{array}\right]$$
  where *a* represents a value of a QoS parameter, $a^{-}$ represents the lower limit interval number, and $a^{+}$ represents the upper limit interval number. When a provider publishes a service, some QoS parameters will fluctuate owing to network instability. The interval can filter out some fluctuations and ensure the authenticity and objectivity of the parameters.
- Step 2: Capture consumer’s requirement.
  Candidate services are selected based on the functional requirements of the user. Users can set various QoS parameters in the form of intervals according to different requirements and additionally set the maximum tolerance threshold for negative parameters. The format is as Equation (4).
  (4) $$\left\{\begin{array}{c} b=\left[\begin{array}{cc} b^{-}, & b^{+} \end{array}\right]\\ Max_{thr}=b^{+}-b^{-} \end{array}\right.$$
  where *b* represents the value of a QoS parameter, $b^{-}$ represents the lower limit interval number, $b^{+}$ represents the upper limit interval number, and $Max_{thr}$ represents the user-acceptable maximum threshold for negative parameters. Considering that the service is dynamic, the appropriate service is reselected if the current dynamic QoS information exceeds the user threshold.
- Step 3: Select candidate services and construct possible degree matrix.
  The QoS parameters of the services requested by consumers are compared with the QoS parameters of the services provided by providers, and then the candidate’s services with the same intersection are selected. Assuming that there are two intervals $a=\left[a^{-},a^{+}\right]$ and $b=\left[b^{-},b^{+}\right]$, and $l_{a}=a^{+}-a^{-}$, $l_{b}=b^{+}-b^{-}$, the formula’s possible degree is calculated by Equation (5) as follows:
  (5) $$P\left(a\geq b\right)=\max\left\{1-\max\left(\frac{b^{+}-a^{-}}{l_{a}+l_{b}},0\right),0\right\}$$
  where P(a≥b) represents the possible degree that *a* is greater than *b*. Calculate the possible degrees and then construct the possible degree matrix. The interval is due to the dynamic variability of the service, and the possible degree is a measure of the distance between the current provider’s service and the consumer’s requirement.
  In this study, TP and SA are positive indicators, whereas RT, BP, and LC are negative indicators; therefore, The Equation (6) is used to express the values corresponding to different indicators.
  (6) $$P=\left\{\begin{array}{c} P\left(c_{ij}\geq d_{ij}\right),\;\mathrm{Negative}\;\mathrm{Indicator}\;\\ P\left(d_{ij}\geq c_{ij}\right),\;\mathrm{Positive}\;\mathrm{Indicator}\; \end{array}\right.$$
  where *P* represents the possible degree, matrix $c_{ij}$ represents the consumer’s requirement intervals, which is the *j*-th index of the *i*-th service, and matrix $d_{ij}$ represents the matrix of the QoS service interval, 1≤i≤n, 1≤j≤m. Suppose that the consumer requirement interval matrix is 13×5; that is, there are 13 candidate services and 5 QoS factors. Therefore, the initial static QoS matrix is a 13×5 matrix. Then, the consumer requirement interval matrix is compared with the static and dynamic QoS parameter matrices of each service, and a 13×5 static possible degree matrix and a 13×5 dynamic possible degree matrix are calculated. The static QoS possible degree matrix is set as the initial evaluation when the service is called for the first time.
- Step 4: Set weight.
  Weight is an important parameter for evaluating multi-attribute decision-making problems. Among the commonly used weight determination methods, the entropy weight method [19] is significantly affected by sample data, which may cause inconsistency with actual cognition, and the analytic hierarchy process [20,21] relies excessively on subjective emotions. In this study, we integrate these two weight determination methods; specifically, the analytic hierarchy process is used to set the subjective weights of the five factors, and the entropy weight method is used to determine the objective weights. Saaty [20] uses a 1–9 scale pairwise comparison method to establish the judgment matrix $a_{jj}\left(1\leq j\leq m\right)$ for QoS parameters and then verifies the consistency of the matrix according to Equations (7) and (8).
  (7) $$CI=\frac{\phi_{\max}-m}{m-1}$$
  (8) $$CR=\frac{CI}{RI}$$
  where CI represents the consistence index, *m* is the order of the matrix, and $\phi_{\max}$ is the maximum eigenvalue of the matrix. When CI=0, it means complete consistency, and the larger CI, the worse the consistency. RI represents the random index, and the corresponding value of RI can be obtained in Table 1. CR represents the consistency ratio. When CR<0.1, the matrix is considered to be valid.
  After verification, the subjective weight is calculated by Equation (9) as follows:
  (9) $$W_{j}^{S}=\frac{\sqrt[{m}]{\prod_{k=1}^{m}a_{jk}}}{\sum \sqrt[{m}]{\prod_{k=1}^{m}a_{jk}}}$$
  where $W_{j}^{S}$ represents the subjective weight and $a_{jk}$ represents the judgment matrix, 1≤j≤m, 1≤k≤m. The objective weight is adjusted according to the change in static or dynamic QoS parameters, whereas the subjective weight is not. Equation (10), which is used to calculate the entropy of the *j*-th index according to the possible degree matrix obtained in Step 3, compares and normalizes the possible degree of each QoS parameter vertically and finally obtains a 1×j matrix. Equation (11) then calculates the objective weights.
  (10) $$e_{j}=\frac{1}{\ln n}\sum_{i=1}^{n}P_{ij}\ln\frac{1}{P_{ij}}$$
  where $e_{j}$ represents the entropy of the *j*-th index and *P* is the possible degree matrix.
  (11) $$W_{j}^{o}=\frac{1-e_{j}}{\sum_{j=1}^{m}1-e_{j}}$$
  where $W_{j}^{o}$ represents the objective weight, $\sum_{j=1}^{m}w_{j}^{o}=1$. Finally, the mixing weight is calculated Equation (12) as follows:
  (12) $$W_{j}=\gamma W_{j}^{s}+\left(1-\gamma \right)W_{j}^{o}\;j=1,2,3,\ldots ,m$$
  where $W_{j}$ represents the mixing weight, $W_{j}^{s}$ represents the subjective weight, $W_{j}^{o}$ represents the objective weight, and γ represents weight ratio, 0≤γ≤1. Using the static possible degree matrix and the dynamic possible degree matrix mentioned above, the static mixing weight and dynamic mixing weight are calculated, respectively.
- Step 5: Calculate global evaluation.
  Finally, we employ the technique for order preference similarity to ideal solutions (TOPSIS) [22,23] to evaluate the service. TOPSIS is a method used to calculate the distance between the candidate service with the best service and the worst service, and then evaluate the service.
  First, build a possible degree evaluation matrix $D=\left\{p_{ij}\right\}$ according to the possible degree interval in Step 3. If there are 13 candidate services and 5 indicators, it is a 13×5 matrix. Then, build a weight matrix $W=\left\{w_{1},w_{2},\ldots w_{m}\right\}$ according to the corresponding mixing weight in Step 4 and rewrite it in diagonal form. There are 5 indicators; therefore, it is a 5×5 matrix. Then, the static or dynamic evaluation matrix is $Z=\left\{z_{ij}\right\}$, where $z_{ij}=p_{ij}*w_{j}$ is a 13×5 matrix to obtain the score of each index of each service. Consequently, the maximum value of each index constitutes a positive ideal solution $z_{j}^{+}$, and the minimum value of each index constitutes a negative ideal solution $z_{j}^{-}$. Finally, according to the calculation of the possible degree of each candidate service, a global evaluation is performed. The service that is closest to the positive ideal solution and farthest from the negative ideal solution is the optimal service. These formulas are shown as Equation (13).
  (13) $$\left\{\begin{array}{c} V_{i}^{+}=\sqrt{\sum_{j=1}^{m}{\left(z_{ij}-z_{j}^{+}\right)}^{2}}\\ V_{i}^{-}=\sqrt{\sum_{j=1}^{m}{\left(z_{ij}-z_{j}^{-}\right)}^{2}}\\ C_{i}=\frac{V_{i}^{-}}{V_{i}^{+}+V_{i}^{-}} \end{array}\right.$$
  where $V_{i}^{+}$ represents the distance of the positive ideal solution, $V_{i}^{-}$ represents the distance of the negative ideal solution, and $C_{i}$ represents the evaluation value of the i-th service. When a service is initialized, it is evaluated according to the static QoS data provided by the service provider. As the number of times the service is invoked increases, the static weight will gradually decrease, and the global evaluation of the service will be more focused on the dynamic evaluation data of the current service. The global evaluation value format is shown as Equation (14).
  (14) $$\left\{\begin{array}{c} Q_{0}=C_{\mathrm{Static}}\\ Q_{\mu }=\frac{1}{\mu +1}Q_{\mu -1}+\frac{\mu }{\mu +1}C_{\mathrm{Dynamic}} \end{array}\right.$$
  where $Q_{0}$ represents the initial value of global evaluation, $C_{\mathrm{Static}}$ represents the static evaluation value, $C_{\mathrm{Dynamic}}$ represents the dynamic evaluation value, μ is the number of times the service has been invoked, and $Q_{\mu }$ is the global evaluation value after μ times.

**Figure 3:**
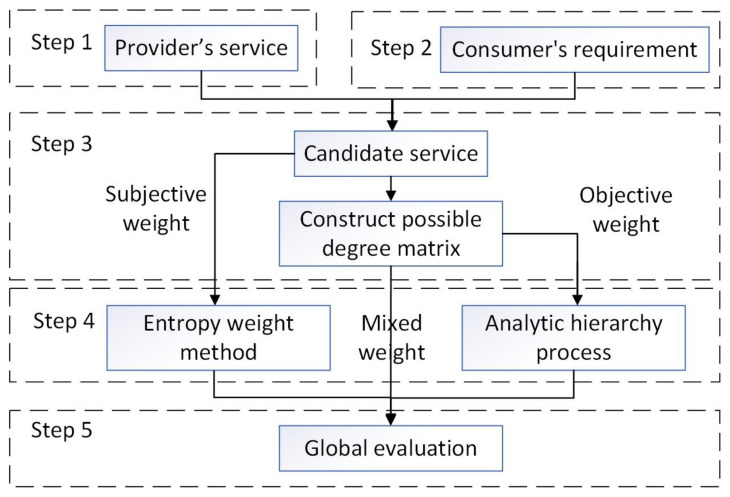
Global evaluation process.

In summary, static QoS evaluation evaluates the initial state of services provided by the participating nodes, and dynamic QoS evaluation evaluates the real-time state of services based on values monitored during the operation of the system. The processes and relationships of these five steps are shown in Figure 3.

## 4. Improved PBFT Consensus Mechanism

### 4.1. Consensus Mechanism

As discussed above, the trust values of the nodes in the system are quantified by QoS-aware trust service evaluation. If a node has a higher global trust value than other nodes, it is considered more trustworthy. We choose nodes with higher trust values to establish the blockchain consensus group. Reducing the number of consensus nodes can improve the efficiency of blockchain consensus algorithms. As the scope of blockchain consensus nodes is narrowed, it can improve the Byzantine fault tolerance rate by selecting nodes with higher trust, thereby reducing the number of messages transmitted and speeding up the process of blockchain consensus consistency. The nodes are divided into two groups based on the global trust values.

#### 4.1.1. Consensus Group

Suppose there are *N* nodes in the network, among which *C* consensus nodes participate in the consensus process. We assume that the number of *C* values can be dynamically adjusted. The format is shown as Equation (15).

(15) C=N×d

where *d* is the constant percentage of nodes. Consensus nodes are responsible for the operation of the entire blockchain system and participate in the voting of the block and chaining process. They receive transactions, work together to determine a consensus, and store the latest state of the public ledger.

#### 4.1.2. Candidate Group

The remaining nodes in the network are candidate nodes and the number is N−C. Nodes in the candidate group do not participate in the consensus, but accept the consensus result. When a Byzantine node appears in the consensus group, it is eliminated after the consensus process. Then, the node with the highest global trust value of the candidate group is selected to join the consensus group to ensure that the nodes in the consensus group are likely to be honest nodes.

#### 4.1.3. Promote–Exclude Mechanism

Only nodes in consensus group are allowed to participate in the following blockchain consensus process, which is described in Algorithm 1 in detail.
**Algorithm 1** conConsensusGroup**Input:** Global trust *T*, Nodes *N*, *d***Output:** ConsensusGroup, CandidateGroup    1: ConsensusGroup = ⊘    2: Sort N by T;    3: **for** $n_{i}\in N$ 
**do**    4:   **if** $T_{i}$ is in the top of *d* **then**    5:     Add $n_{i}$ into ConsensusGroup;    6:   **else**    7:     Add $n_{i}$ into CandidateGroup;    8:   **end if**    9: **end for**  10: **Return** ConsensusGroup, CandidateGroup

In the initialization stage, *N* blockchain nodes are sorted in descending order according to the QoS evaluation value *T* of the service. The nodes at the top of *d* are selected to form the consensus group *C*, and the remaining nodes join the candidate group. The promote–exclude mechanism then establishes a dynamic balance between the consensus and candidate groups. After the consensus group is constructed, the algorithm will continue to perform the optimized consensus process. When a consensus cycle is completed, the consensus and candidate groups are updated based on the latest QoS global trust value of the node. Nodes with poor performance in the consensus group, that is, nodes with lower QoS value *T*, are excluded from the consensus group, and nodes with the highest QoS value in the candidate group are promoted to the consensus group. When a normal node becomes a Byzantine node and is discovered, it is punished by QoS value reduction and then promptly removed from the consensus group. New nodes with high QoS values can also become consensus nodes. In this way, it can reduce the number of consensus nodes, speed up the blockchain consensus process, and significantly improve the dynamics and robustness of the network.

### 4.2. Consensus Process

**Figure 4:**
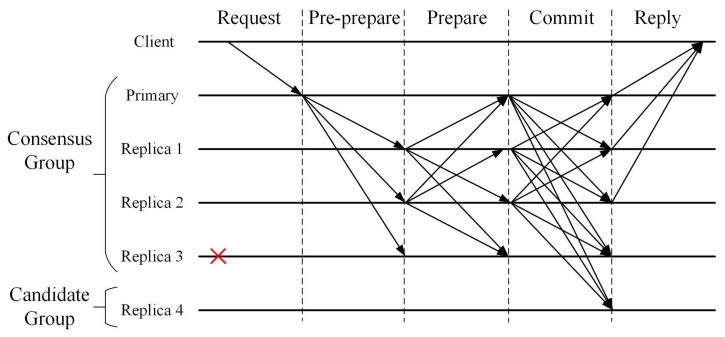
The optimized consensus process.

The PBFT consensus algorithm reaches a consensus on a single operation between *n* servers, which requires extensive calculation and communication, such as exchanging messages, leading to $O(N^{2})$ complexity. Assuming that in a network, the total number of nodes is *N* and the number of malicious nodes is *f*, the normal network operation needs to satisfy N≥3f+1. Under this assumption, it can be guaranteed that the operation of the system will not be stopped because of the influence of malicious nodes. Regardless of the 3f+1 nodes in a distributed system, where *f* represents the number of Byzantine nodes, the system can survive *f* fault nodes and can reach agreement as long as there are no less than 2f+1 non-Byzantine nodes. The optimized consensus protocol combined with the improved consensus group is shown in Figure 4.

The improved PBFT consensus rounds are broken into five phases below:

(1) Request phase. In this phase, a client sends a request message to the primary node in the network. The format of the request message is shown as Equation (16).
  (16) <Request,o,t,c>
  where Request contains message details *m* and message digest d(m), *o* represents the requested operation, *t* represents the timestamp, and *c* represents the client ID.
(2) Pre-prepare phase. When the primary node receives the request, it enters the pre-prepare phase, and announces the next record that the consensus group should agree to, which is realized by sending a pre-prepare message. The primary node sorts the transaction requests, assigns a number *n*, and generates a pre-prepare message, which is broadcast to other replica nodes. The format of a pre-prepare message is shown as Equation (17).
  (17) <<Pre−prepare,v,n,d,h>,g,m>
  where *v* represents the view number, *d* represents the message digest of the client, *m* represents the message details, *g* is the global trust value of node, and *h* is the result of hash calculation on *g*.
(3) Prepare phase. After each node in the consensus group receives the pre-prepare message, it verifies the correctness and validity of the record, and determines whether the *h* value in the pre-prepare message is the same as the local *h* value. If they are different, the local global trust value will be updated to *g*. Then a prepare message is multicast to all the other nodes. The format of the prepare message is shown as Equation (18).
  (18) <Prepare,v,n,d,i>
  where *i* represents the current replica node ID. The prepare phase is complete when a replica node obtains 2f valid prepared messages from different replica nodes, where *f* is the number of Byzantine nodes in the system.
(4) Commit phase. If replica node *i* receives 2f+1 verified PREPARE messages, it will send commit messages to other nodes, including the primary node. After receiving the prepare messages from the 2/3 majority, the primary node multicasts a commit message to both the consensus group and the candidate group. The format of the commit message is shown as Equation (19).
  (19) <Commit,v,n,d,i>
  At this stage, the primary node will receive feedback messages from all consensus nodes and verify the validity of the messages. Once the transaction information *m* is tampered with, its hash value *d* will be changed accordingly. If the value of *d* is different, it means that the transaction message has been tampered with, so it is determined that the node sending the feedback message is a Byzantine node. When a Byzantine node is identified, the node will be penalized, i.e., the QoS global trust value will drop by 50%. Finally, each node waits for more than 2/3 of commit messages from the consensus group to ensure that a sufficient number of nodes agree with the record proposed by the leader.
(5) Reply phase. In this phase, the client waits for f+1 replies from different nodes in the consensus group. The results of these replies should be the same, where *f* represents the maximum number of potentially faulty nodes. The format of the commit message is shown as Equation (20).
  (20) <Reply,v,t,c,i,r>
  where *r* represents the result of the request. If the client receives f+1 identical reply messages, it means that the request has reached a consensus on the entire network.

## 5. Analysis and Evaluation

### 5.1. Qos-Aware Trust Service Validation

#### 5.1.1. Dataset and Selected Services

We evaluated the performance of the QoS-aware trust service global evaluation mechanism based on a real dataset: the Quality of Web Service (QWS) dataset [24,25] Version 2.0, (last updated: 1 November 2019). The QWS dataset includes a set of 2507 web services and their QWS measurements. Each web service has nine corresponding QWS measurements, which were measured using multiple web service benchmark tools over a six-day period. The QWS values represent the average of the measurements collected during that period. We selected 13 testing query services for the evaluation, which should be similar services invoked in the same domain, and selected five QoS indicators, namely, RT, TP, SA, BP, and LC, to verify the feasibility of the proposed model. RT, BP, and LC are negative indicators and TP and SA are positive indicators. Table 2 lists the QoS indicator values for the selected services.

#### 5.1.2. Results of Global Value

We calculated the global value of each service according to the user’s requirements to select the most suitable service. Assume a user requirement as shown in Table 3. The user wishes to select the best service among these 13 services. Broadly speaking, the smaller the value of the negative indicator, the better the service performance. Therefore, a user-acceptable maximum threshold requirement is set for each negative indicator. Conversely, the larger the value of the positive indicator, the better the service performance; thus, there is no maximum threshold limit.

The subjective weight is calculated using Equation (9), and the weight will not change whether it is static or dynamic. Then, the static and dynamic objective weights are calculated using Equations (10) and (11), respectively. The static possible degree and the dynamic possible degree are calculated using Equations (5) and (6), respectively. Finally, the mixing weight is calculated using Equation (12) and the ratio of the subjective and objective weights are set considering the user’s requirements. The values of various weights are shown in Table 4.

The evaluation value for each service can be obtained using Equation (13). Where the values of CSP7, CSP8, and CSP11 are higher than the user’s threshold and do not meet the user’s requirements; therefore, they are rejected. The static, dynamic, and global value evaluation results for services that meet user requirements are shown in Table 5.

It can be seen that CSP10 is the best service, with a global value of 0.81, followed by CSP1. From Equation (14), we know that the global value is calculated using static and dynamic values. As can be observed in Table 5, similar to CSP9, the dynamic QoS value of 0.53 is higher than the static value of 0.38, so the global value of the service is also increased. By contrast, similar to CSP1, the dynamic QoS value of 0.63 is lower than the static value of 0.78; therefore, the global value of the service is also reduced. This indicates that the global value of the service changes dynamically with the dynamic QoS value, reflecting the real-time QoS performance status of the service. As the number of services invoked increases, the weight of the dynamic values increases. The proposed evaluation strategy tends to reflect the latest dynamic QoS in real time.

#### 5.1.3. Effects of Parameter Adjustment

We selected the top five global values of services (CSP10, CSP1, CSP5, CSP13, and CSP2), adjusted three parameters (dynamic QoS parameter, user requirements, and subjective weight), and observed the results of the evaluation model.

**Figure 5:**
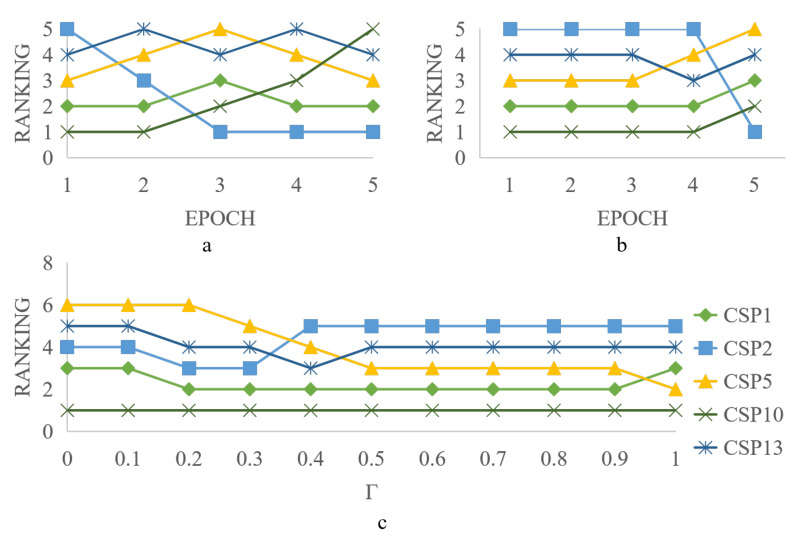
Changes in global value ordering under different parameter adjustments. (**a**) QoS parameters. (**b**) User requirements. (**c**) Subjective weight coefficient γ.

First, we set γ=0.5, degraded the QoS parameters of the No. 1 service CSP10, and optimized the QoS parameters of the No. 5 service CSP2 to judge whether the proposed scheme can correctly reflect the service changes in real time. The experimental results are presented in Figure 5a. It can be seen that CSP2, which was the worst service, has transformed into the best service by continuously acquiring high-quality dynamic QoS parameters, whereas CSP10 has changed from the best service to the worst service owing to degraded dynamic QoS parameters. Therefore, the evaluation can accurately reflect service performance in real time.

Second, we set γ=0.5, when the user requirements are constantly changing, the global value rankings of CSP1, CSP2, CSP5, CSP10, and CSP13 are shown in Figure 5b. It can be seen that the global value ranking changes when the user demands are constantly changing. The global value is calculated from the static and dynamic QoS values according to Equation (14). The static QoS value remains unchanged, and the dynamic QoS value changes when the user demands are constantly changing, similar to the global trust value. Therefore, it can be concluded that user demand affects global value. As user requirements change, the global value ranking also changes.

Finally, we set γ={0,0.1,0.2,…,0.9,1}, when γ is changing, the global value ranking of CSP1, CSP2, CSP5, CSP10, and CSP13 is shown in Figure 5c. It can be seen that with the change in γ, the global value ranking of services constantly changes. Therefore, it can be concluded that the subjective weight coefficient affects service ranking. These results indicate that the proposed method effectively and accurately reflects service changes in real time.

### 5.2. Efficiency Evaluation

Experimental studies are performed on UOS v20. The computer has an Intel^®^ Core^™^ i7 CPU 10750H processor and 8 GB RAM. We implement a multi-node blockchain experimental system based on Java, and evaluate the proposed QTPBFT algorithm with an original PBFT algorithm from three dimensions: communication complexity (CC), transaction latency (TL), and transaction throughput (TT).

#### 5.2.1. Communication Complexity

Communication complexity is the amount of communication required to complete the consensus process. Assuming that there are *n* nodes in the blockchain system. In the traditional PBFT algorithm, all nodes participate in the consensus process. A single process has three stages: pre-prepare, prepare, and commit. The number of communications is n−1, n×(n−1), and n×(n−1), respectively. That is, a one-to-all broadcast and two all-to-all broadcasts. Therefore, the total number of communications in a single consensus process for PBFT $Count_{PBFT}$ is calculated by Equation (21) as follows:

(21) $$Count_{PBFT}=n-1+n\times \left(n-1\right)+n\times \left(n-1\right)=2n^{2}-n-1$$

where *n* represents the number of nodes in the blockchain system.

**Figure 6:**
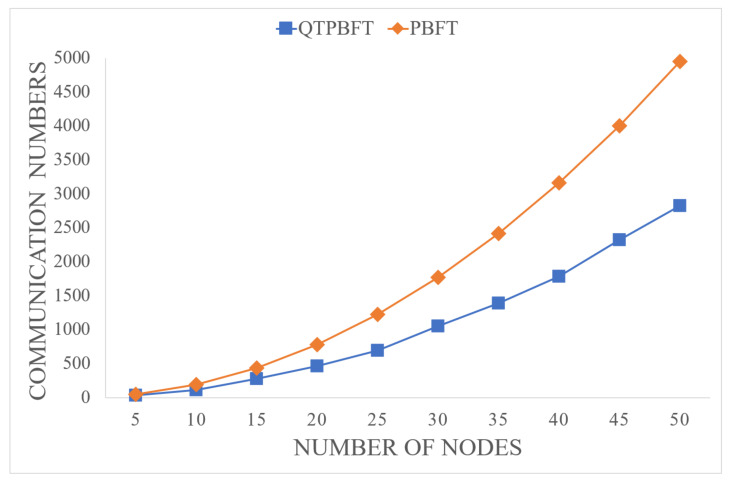
Comparison of communication numbers for a single consensus of the two algorithms.

In our proposed QTPBFT, only selected nodes, that is, top d(0<d⩽1) nodes with the highest trust value, participate in the consensus process, rather than all nodes in the network. To ensure the consensus operation, the maximum number of nodes in the candidate group should be $\left\lfloor \frac{n-1}{3}\right\rfloor$, so the number of nodes in the consensus group is calculated by Equation (22) as follows:

(22) $$n^{\prime }=n-\left\lfloor \frac{n-1}{3}\right\rfloor$$

where *n* represents the number of nodes and $n^{\prime }$ represents the number of nodes in the consensus group. Then, we can calculate *d* by Equation (23) as follows:

(23) $$d=\frac{n^{\prime }}{n}=\frac{n-\lfloor \frac{n-1}{3}\rfloor }{n}=\frac{2}{3}+\frac{1}{3n}$$

where *n* represents the number of nodes. The value of *d* tends to be in the range of $\left[\frac{2}{3}+\frac{1}{3n},1\right]$; that is, consider the worst case, when d=1, it will degenerate into a PBFT process. The smaller the value of *d*, the lower the communication complexity. Obviously, there will be fewer messages in the consensus process. The communication complexity has been narrowed down overall. Therefore, the total number of communications in a single consensus process for QTPBFT is calculated by Equation (24) as follows:

(24) $$Count_{QTPBFT}=n^{\prime }-1+n^{\prime }\times \left(n^{\prime }-1\right)+n^{\prime }\times \left(n-1\right)=\frac{10}{9}n^{2}+\frac{1}{9}n-\frac{11}{9}$$

where $n^{\prime }$ represents the number of nodes in the consensus group. Comparing Equations (21) and (24), it can be concluded that the number of communications of QTPBFT is much smaller than that of PBFT. Figure 6 compares communication numbers for a single consensus of QTPBFT and PBFT systems with the same number of nodes.

It can be seen that with an increasing number of nodes in the system, the communication numbers for a single consensus of the QTPBFT algorithm increase linearly, whereas the communication numbers for a single consensus of the PBFT algorithm increase exponentially. The communication complexity of QTPBFT is much lower than PBFT. It can be concluded that the QTPBFT algorithm reduces the communication complexity of the network significantly.

#### 5.2.2. Transaction Latency

Transaction latency is the time required for the blockchain platform to respond to the transaction. It takes the time from when a node initiates a transaction request to when the entire transaction process is completed. Lower transaction latency means the system can acknowledge messages faster and with higher throughput. Therefore, the transaction latency is an important indicator to measure blockchain performance. Its calculation formula is calculated by Equation (25) as follows:

(25) $$delay=t_{finish}-t_{request}$$

where $t_{finish}$ represents the time when the transaction is completed, $t_{request}$ represents the transaction request time.

**Figure 7:**
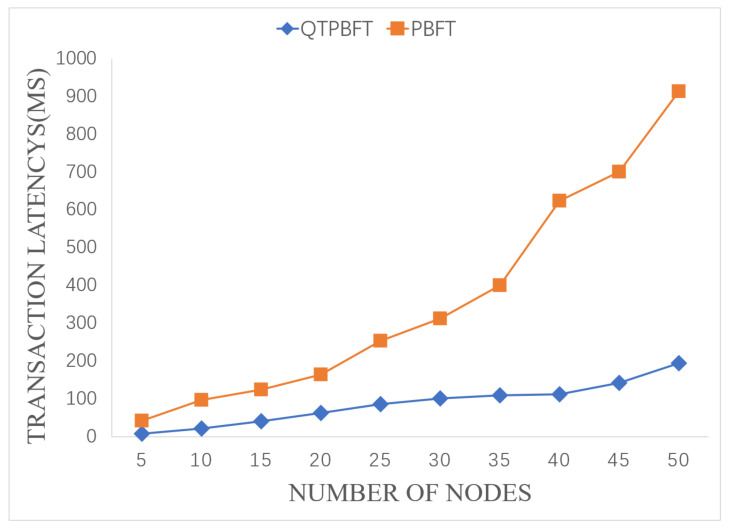
Comparison of the transaction latency of the two algorithms.

In the proposed blockchain system, large-scale transactions between service providers and consumers become more complicated. There are multiple participants communicating for the transaction, waiting for the next block to be verified across multiple nodes. The blockchain key management, identity certification, and digital signatures will also add a slight latency. Figure 7 shows the comparison of transaction latency of QTPBFT and PBFT systems with the same number of nodes.

It can be observed from Figure 7 that, as the number of participants increases, the transaction latency of the system will also increase. However, transaction latency of the QTPBFT algorithm is significantly better than the PBFT algorithm. When the node number increases to 50, the latency of QTPBFT processing a transaction is 195 ms, which is much smaller than the 913 ms of PBFT. The transaction latency of the PBFT algorithm increases rapidly, whereas the transaction latency of the QTPBFT algorithm is more stable and increases slowly.

#### 5.2.3. Transaction Throughput

Transaction throughput is also another important metric for measuring consensus algorithms. Transaction throughput represents the number of transactions that the network can process per second, that is, the number of transactions generated over time during the test. Throughput performance is usually expressed as transactions per second (TPS) when presenting performance test results. Its calculation formula is calculated by Equation (26) as follows:

(26) $$TPS=\frac{\;\mathrm{transactions}\;}{\Delta t}$$

where transactions is the number of transactions written to the blockchain. Δt is the time interval from transaction occurrence to confirmation.

**Figure 8:**
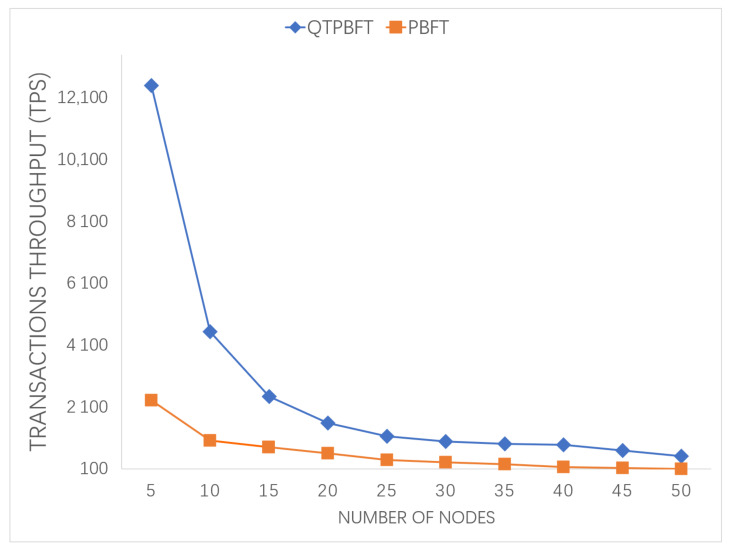
Comparison of the transaction throughput of the two algorithms.

Figure 8 shows the comparison of transaction throughput of QTPBFT and PBFT systems with the same number of nodes.

It can be observed that, as the number of nodes in the network increases, the transaction throughput of both algorithms will decrease. However, the transaction throughput of the QTPBFT algorithm is much higher than that of PBFT algorithm.

### 5.3. Comparison with Other Optimization Mechanisms

The QTPBFT optimization mechanism proposed in this paper is compared and analyzed with other methods reported for the consensus optimization mechanism. From the literature survey, we show that the proposed approach achieves better results than all the previous methods to the best of our knowledge. A brief description of these methods and the comparison results are shown in Table 6.

The proposed QTPBFT narrows the consortium nodes to a consensus group with higher trust values. Theoretically, the number of messages delivered among nodes is $O\left(d\times N^{2}\right)\left(0<d⩽1\right)$. For participating nodes, we score them based on their performance, then sort and group nodes, which is different from most consensus algorithms. We use the consensus group instead of individual nodes to enhance robustness. This can not only ensure the privacy of the consensus group nodes and reduce the probability of the primary node’s Byzantine behavior by mutual supervision, but also reduce the probability of the view change process. However, specific to practical scenarios, different scenarios have different demands and different consensus protocols should be adopted to provide the corresponding optimal performance. As such, compared with other optimized consensus algorithms, the proposed QTPBFT reduces consensus nodes through a QoS-aware trust value mechanism, providing low complexity and high scalability. Moreover, the trust value of the consensus nodes is dynamically adjusted according to the status of the service, which is more suitable for real-time service-transaction scenarios.

## 6. Conclusions

In this paper, we propose an improved consensus algorithm, QTPBFT, which is based on the QoS-aware trust service. It incorporates a QoS-aware trust service global evaluation mechanism to ensure that nodes with higher trust values are more trustworthy and that the global trust value of the nodes is updated dynamically. Further, it forms the consensus group based on the trust value mechanism, which significantly reduces the number of consensus nodes and the number of messages to be transmitted to guarantee that all nodes participating in the consensus algorithm are trusted nodes, thereby reducing the complexity of communication and improving the efficiency of consensus. A practical protocol is also introduced for the QTPBFT system, and the format of the broadcast message is designed to ensure that it can work in peer-to-peer networks, adjust the trust value adaptively, and reach consensus efficiently. Theoretical analysis and simulation experiments indicate that the proposed QTPBFT can dynamically adjust the trust value according to the performance of the node and improve the consensus efficiently.

## Figures and Tables

**Table 1:** The standard values of RI.

Order of Matrix	1	2	3	4	5	6	7	8	9	10
RI	0	0	0.58	0.90	1.12	1.24	1.32	1.41	1.45	1.49

**Table 2:** The dynamic QoS indicator values for 13 selected services.

Service ID	Service Name	RT	TP	SA	BP	LC
CSP1	GoogleSearchService	133	7.7	95	84	10.67
CSP2	DiscoveryService	134.07	12.2	85	69	8.21
CSP3	CSearch	184.67	2.7	74	80	40.84
CSP4	AddressLookup	141.77	7.5	56	77	88.31
CSP5	SearchService	151.33	6.9	99	84	8.66
CSP6	AmazonSearchService	47.27	20.3	62	82	2
CSP7	AddressFinder	203.57	1.2	59	72	110.5
CSP8	redataService	383.2	2.1	100	84	6.2
CSP9	SearchCuroCustomerService	171	18.6	84	87	5
CSP10	search	58	16	98	80	1
CSP11	findkmService	204.6	1.9	99	75	7.8
CSP12	LookingForStrategyServices	149.67	11.2	95	84	82
CSP13	GoogleSearchServiceTwo	121	7.9	97	84	10

**Table 3:** User requirement.

	RT	TP	SA	BP	LC
Threshold	200	—	—	100	100
Requirement	[100, 150]	[5, 20]	[80, 100]	[50, 80]	[5, 50]

**Table 4:** The values of various weights.

Name	RT	TP	SA	BP	LC
Subjective weight	0.09	0.05	0.42	0.23	0.21
Static QoS objective weight	0.24	0.11	0.27	0.13	0.25
Dynamic QoS objective weight	0.24	0.17	0.21	0.22	0.16
Static QoS mixing weight	0.17	0.08	0.34	0.18	0.23
Dynamic QoS mixing weight	0.17	0.11	0.32	0.22	0.18

**Table 5:** The static value, dynamic value, and global value evaluation result of services.

Service ID	Static Value	Dynamic Value	Global Value
CSP1	0.78	0.63	0.7
CSP2	0.7	0.61	0.65
CSP3	0.28	0.15	0.21
CSP4	0.28	0.18	0.23
CSP5	0.74	0.6	0.67
CSP6	0.53	0.55	0.54
CSP9	0.38	0.53	0.45
CSP10	0.77	0.85	0.81
CSP12	0.47	0.44	0.46
CSP13	0.66	0.67	0.66

**Table 6:** Comparison results of the proposed method with other methods.

Consensus	Byzantine Fault Tolerance	Communication Complexity	Node	View Change Probability
Original PBFT [9]	Yes	$O(N^{2})$	Single	High
Zyzzyva [10]	Yes	O(N)	Single	High
SBFT [11]	Yes	$O(N^{2})$	Single	High
RBFT [14]	No	O(N)	Single	High
T-PBFT [16]	Yes	$O(N^{2})$	Group	Low
X-layer PBFT [17]	Yes	$1.9N^{\frac{4}{3}}$	Multi-Layer	High
Proposed method	Yes	$O(d\times N^{2})$	Group	Low

## Data Availability

Publicly available dataset the QWS Dataset was analyzed in this study. This data can be found here: https://qwsdata.github.io/.
